# Induction chemotherapy with paclitaxel, carboplatin, and cetuximab (PCE) followed by chemoradiotherapy for unresectable locoregional recurrence after curative surgery in patients with squamous cell carcinoma of the head and neck

**DOI:** 10.3389/fonc.2024.1420860

**Published:** 2024-07-01

**Authors:** Masanobu Sato, Tomohiro Enokida, Takao Fujisawa, Susumu Okano, Naohiro Takeshita, Nobukazu Tanaka, Hideki Tanaka, Atsushi Motegi, Sadamoto Zenda, Takeshi Shinozaki, Kazuto Matsuura, Ryuichi Hayashi, Tetsuo Akimoto, Makoto Tahara

**Affiliations:** ^1^ Department of Head and Neck Medical Oncology, National Cancer Center Hospital East, Kashiwa, Japan; ^2^ Department of Head and Neck Surgery, National Cancer Center Hospital East, Kashiwa, Japan; ^3^ Department of Otorhinolaryngology, Graduate School of Medical Sciences, Kyushu University, Fukuoka, Japan; ^4^ Department of Otorhinolaryngology, Jikei University School of Medicine, Tokyo, Japan; ^5^ Department of Radiation Oncology and Particle Therapy, National Cancer Center Hospital East, Kashiwa, Japan

**Keywords:** unresectable squamous cell carcinoma of the head and neck, induction chemotherapy, PCE, cetuximab, chemoradiotherapy

## Abstract

**Background:**

The significance of induction chemotherapy (IC) in the treatment of squamous cell carcinoma of the head and neck (SCCHN) with unresectable locoregional recurrence after curative surgery has not been clarified. The aim of this study was to evaluate the efficacy of IC followed by chemoradiotherapy (CRT) in these patients.

**Methods:**

Among patients with unresectable locoregional recurrent SCCHN who had not undergone prior irradiation and were eligible for cisplatin, we conducted a retrospective analysis of patients who received CRT following IC with paclitaxel, carboplatin, or cetuximab (IC-PCE group) and those who received CRT without prior IC (CRT group) between June 2013 and August 2021.

**Result:**

Forty-two patients were included. The CRT group and IC-PCE group consisted of 15 and 27 patients, respectively. Primary site was the oral cavity (n=25), oropharynx (n=3), hypopharynx (n=13) and larynx (n=1). Objective response rate (ORR) with IC-PCE was 55.6%; 24 patients (88.9%) subsequently received CRT. ORR after completion of CRT was significantly better in the IC-PCE group (95.8% in the IC-PCE group vs. 66.7% in the CRT group, p=0.024). Progression-free survival (PFS) of the total population on median follow-up of 2.4 years (range: 0.8-7.3) tended to be better in the IC-PCE group (2-year PFS: 55.6% in the IC-PCE group vs. 33.3% in the CRT group, log-rank *p*=0.176), especially in oral cancer (2-year PFS: 37.5% in the IC-PCE group vs. 0% in the CRT group, log-rank *p*=0.015).

**Conclusion:**

Therapeutic strategies including IC-PCE in patients with unresectable locoregional recurrent SCCHN after curative surgery may contribute to improved prognosis, especially in oral cancer.

## 1 Introduction

Squamous cell carcinoma of the head and neck (SCCHN) occurs mainly in the oral cavity, pharynx, and larynx, and globally accounts for 8% of all epithelial malignancies. Approximately 60% of patients present with advanced disease (1). Locally advanced SCCHN (LA-SCCHN) is usually treated multimodally with surgery and radiotherapy or concurrent chemoradiotherapy (CRT), but local recurrence occurs at a rate of 50-60%.

Treatment of local recurrence after curative therapy is determined by the feasibility of surgical resection and the presence or absence of prior radiation therapy; according to NCCN guidelines, in the absence of prior irradiation, resection of the recurrent lesion ± postoperative adjuvant therapy (radiation therapy alone or concurrent CRT) is offered if applicable. However, when surgical resection is performed as prior curative treatment, especially with reconstruction procedures, further radical resection is often technically challenging. Also, some cases of recurrence are rapidly progressive, a situation which also hampers surgical intervention; examples include early locoregional recurrence detected by planned computed tomography (CT) or 18-fluorodeoxyglucose positron emission tomography with CT (PET/CT) performed for postoperative radiotherapy for patients at risk of recurrence after radical resection (2, 3). Overall, the percentage of patients able to receive salvage surgery for loco-regional recurrent lesions is 27-65%. Moreover, prognosis in patients who do not undergo surgical salvage but receive radiation therapy and chemotherapy is dismal, with one group reporting a mean survival of 7 months (4). A similarly poor prognosis is also reported for non-surgical treatment, including chemoradiotherapy, with a 3-year OS of 9-10% in patients with local recurrence of oral cancer after initial surgery (5, 6). These findings highlight the critical need for reliable non-surgical therapeutic strategies for this patient population.

Induction chemotherapy (IC) in head and neck cancer is generally defined as systemic chemotherapy performed prior to concurrent CRT as an initial curative therapy aimed at improving the survival prognosis. Historically, the combination of docetaxel, cisplatin (CDDP), and 5-fluorouracil (TPF) has been used and tested in this setting (7–10). To our knowledge, however, no study has investigated the significance of IC itself amongst patients with locoregional recurrence which is not suitable for surgical intervention. In this situation, we have adopted IC consisting of paclitaxel (PTX), carboplatin (CBDCA), and cetuximab (IC-PCE) followed by CRT (11) for patients with SCCHN who develop locoregional recurrence but have no prior irradiation history. IC-PCE prior to curative therapy is feasible and effective (12), and is recommended as a category 2B induction chemotherapy regimen in the NCCN guidelines. To date, however, its use in patients with recurrence has not been evaluated.

In this study, we assessed the potential clinical significance of this therapy by a comprehensive review of patients treated in our institution.

## 2 Materials and methods

### 2.1 Patients

We retrospectively reviewed the medical records of 53 patients with unresectable locoregional recurrent SCCHN treated with radiotherapy from June 2013 to August 2021 at the National Cancer Center Hospital East in Japan. Of these patients, those who met all the following eligibility criteria were selected as the target population: primary site of origin in the oral cavity, oropharynx, hypopharynx, or larynx; recurrence after curative resection; no evidence of distant metastasis; and planned to receive either IC-PCE followed by CRT consisting of cisplatin (CDDP) and RT or CRT alone. Unresectable locoregional recurrence was defined as meeting any of the following: (i) difficulty in surgical resection with enough surgical margin (e.g., carotid artery invasion, skull base invasion, prevertebral muscle invasion, and unreconstructible); or (ii) inability to control tumor progression by surgical resection (e.g., early and rapidly progressed locoregional recurrence revealed by planned CT before postoperative radiotherapy, and far advanced lymph node metastasis [retropharyngeal so-called “Rouviere”, mediastinal or supraclavicular lymph nodes]).

The decision to offer IC-PCE followed by CRT or upfront CRT was primarily determined in a multidisciplinary meeting in which medical oncologists, radiation oncologists, diagnostic radiologists, and head and neck surgeons discussed individual patients. Patients who received radiotherapy alone, who were intolerant to CDDP, who received induction chemotherapy other than PCE, or who had synchronous multiple cancers were excluded (**Supplementary Figure 1**). This study was approved by the Institutional Review Board of the National Cancer Center Hospital East (2016-243).

### 2.2 Treatment

The induction PCE regimen consisted of CBDCA area under the plasma concentration-time curve (AUC) = 1.5, PTX 80 mg/m^2^, and cetuximab at an initial dose of 400 mg/m^2^ followed by 250 mg/m^2^ administered weekly for eight weeks. Following IC, concurrent CRT was started. During CRT, CDDP was administered intravenously at a dose of 80 mg/m^2^ every three weeks on days 1, 22, and 43 (Tri-CDDP) or at a dose of 20 mg/m^2^ on days 1-4, repeated three times at 3-week intervals (Fractionated tri-CDDP). As with radiotherapy, all patients were treated with intensity-modulated radiation therapy (IMRT). The planned total radiation dose was 70 Gy (2Gy per day, five days per week). Toxicity during treatment was graded using the Common Toxicity Criteria for Adverse Events (CTCAE version 5.0).

### 2.3 Evaluation of efficacy and statistical analysis

Clinical tumor response was evaluated according to the Response Evaluation Criteria in Solid Tumors (RECIST) v.1.1 via the review of computerized tomography (CT) or magnetic resonance (MRI) imaging and [18F]-fluorodeoxyglucose positron-emission tomography (PET)/CT. The first post-CRT tumor response assessment was performed 8–12 weeks (2-3 months) after the end of radiotherapy using a CT scan or MRI in all cases, considering the potential delayed tumor regression. In addition, as for the IC-PCE group, the evaluation for tumor response by IC-PCE was performed within 1-2 weeks of the last cycle performed. Optionally, an interim evaluation was planned when completing four cycles of PCE. Patients were continuously investigated for tumor progression and survival until death, loss to follow-up, or end of the cutoff period, whichever occurred first. Overall survival (OS) was defined as the time from the initiation of treatment to death for any reason. Progression-free survival (PFS) was defined as the time from initiation of treatment to initial disease recurrence, progression of disease, or death from any cause. OS and PFS were estimated by the Kaplan–Meier method (log-rank test). For patients who were treated with CRT, the proportion with CRT completion (%CRT completion) was also evaluated, defined by (a) completion of a cumulative CDDP dose ≥ 200mg/m^2^ and (b) completion of radiotherapy within two weeks after the planned completion date.

All statistical analyses were performed with EZR (version.1.51; Saitama Medical Center, Jichi Medical University, Saitama, Japan), which is a graphical user interface for R (The R Foundation for Statistical Computing, Vienna, Austria; v. 4.1.1). More precisely, it is a modified version of R commander which was designed to add statistical functions frequently used in biostatistics (13).

## 3 Results

### 3.1 Patients characteristics

Of the 53 patients with unresectable locoregional recurrence after curative surgery and no previous irradiation, 42 patients who were planned to receive either CDDP + RT (CRT group, n=15) or IC-PCE followed by CDDP +RT (IC-PCE group, n=27) at the initiation of treatment were identified (**Supplementary Figure 1**). **Table 1** and **Supplementary Table 1** compare the main patient characteristics between the CRT group and IC-PCE group. There was no statistically significant difference in clinical background, except that the IC-PCE group had a higher proportion of patients with hypopharynx primary disease and advanced pathologically proven lymph node metastasis at the most recent surgery.

**Table 1:** Patient characteristics.

	CRT group (n=15)	IC-PCE group (n=27)	*p*-value
Age			
Median (range)	58 (18-76)	65 (27-74)	0.498
Gender (%)			
Male	9 (60.0)	20 (74.1)	0.488
Female	6 (40.0)	7 (25.9)	
Primary (%)			
Oral	9 (60.0)	16 (59.3)	0.011
Oropharynx	3 (20.0)	0 (0)	
Hypopharynx	2 (12.3)	11 (40.7)	
Larynx	1 (6.7)	0 (0)	
Smoking (%)			
Never	6 (40.0)	8 (29.6)	0.849
Former	4 (26.7)	9 (33.3)	
Current	5 (33.3)	10 (37.0)	
Alcohol (%)			
Never	6 (40.0)	6 (22.2)	0.559
Occasional	2 (13.3)	5 (18.5)	
Every day	7 (46.7)	16 (59.3)	
pStage at initial surgery (%)			
I	1 (6.7)	2 (7.4)	0.678
II	2 (13.3)	2 (7.4)	
III	3 (20.0)	2 (7.4)	
IVA	5 (33.3)	10 (37.0)	
IVB	4 (26.7)	11 (40.7)	
Neck dissection at initial surgery (%)			
None	3 (20.0)	4 (14.8)	0.453
Ipsilateral	5 (33.3)	5 (18.5)	
Bilateral	7 (46.7)	18 (66.7)	
Type of most recent surgery (%)			
Initial surgery	11 (73.3)	23 (85.2)	0.425
Surgery for recurrent lesions (salvage surgery)	4 (26.7)	4 (14.8)	
pT stage at the most recent surgery (%)			
T1	0 (0)	1 (3.7)	0.302
T2	2 (13.3)	1 (3.7)	
T3	3 (20.0)	3 (11.1)	
T4a	6 (40.0)	17 (63.0)	
T4b	0 (0)	1 (3.7)	
rTx	3 (20.0)	1 (3.7)	
rT1	1 (6.7)	0 (0)	
rT2	0 (0)	1 (3.7)	
rT3	0 (0)	1 (3.7)	
rT4a	0 (0)	1 (3.7)	
pN stage at the most recent surgery (%)			
Nx	1 (6.7)	0 (0)	0.046
N0	4 (26.7)	4 (14.8)	
N1	0 (0)	7 (25.9)	
N2a	0 (0)	1 (3.7)	
N2b	2 (13.3)	0 (0)	
N2c	0 (0)	1 (3.7)	
N3b	4 (26.7)	10 (37.0)	
rNx	0 (0)	1 (3.7)	
rN1	3 (20.0)	1 (3.7)	
rN2b	1 (6.7)	2 (7.4)	
Surgical margin status at the most recent surgery (%)			
Positive/close	0 (0)	6 (22.2)	0.149
Negative	12 (80.0)	20 (74.1)	
No resection of primary site	3 (20.0)	1 (3.7)	
ECE status at the most recent surgery (%)			
Positive	6 (40.0)	11 (40.7)	0.807
Negative	5 (33.3)	12 (44.4)	
No metastasis	3 (20.0)	3 (11.1)	
No neck dissection	1 (6.7)	1 (3.7)	
Recurrence-free interval (RFI) from the most recent surgery			
Median (range)	104 (20-300)	55 (15-391)	0.460
≤2 months (%)	6 (40.0)	14 (51.9)	0.329
2<, ≤6 months (%)	6 (40.0)	5 (18.5)	
6 months < (%)	3 (20.0)	8 (29.6)	
Recurrence site after the most recent surgery (%)			
Local	5 (33.3)	5 (18.5)	0.592
Regional	9 (60.0)	16 (59.3)	
Loco-regional	1 (6.7)	5 (18.5)	
Mediastinal lymph node	0 (0)	1 (3.7)	

RT, radiotherapy; IC, induction chemotherapy; ECE, extracapsular extension.

### 3.2 Treatment delivery and outcome

**Figure 1:**
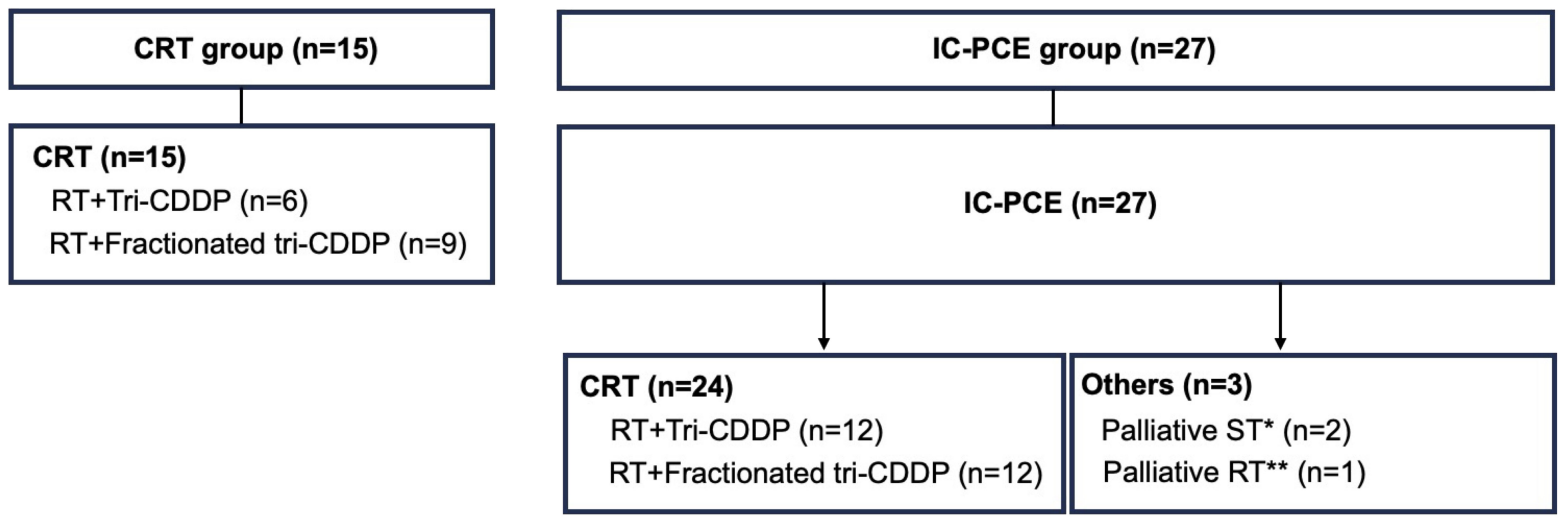
Details of treatment delivery in the CRT and IC-PCE groups. ^*^Two patients received palliative chemotherapy as subsequent systemic therapy for progressive disease. ^**^One patient received palliative RT to prevent skeletal-related events due to bone metastases after PCE failure. CRT, chemoradiotherapy; RT, radiotherapy; tri-CDDP, tri-weekly cisplatin; IC, induction chemotherapy; PCE, paclitaxel + carboplatin + cetuximab; ST, systemic therapy.

**Figure 1** shows the actual treatment delivery in this patient cohort. In the CRT group, all patients were treated with CRT without prior administration of IC. In the IC-PCE group, patients received IC-PCE as initial treatment, followed by subsequent therapy according to tumor response as well as the patient’s condition at the completion or termination of IC-PCE.

In the IC-PCE group, the median number of administered PCE cycles was seven (range, 1-8), and 13 patients (48.1%) completed the eight cycles of IC as planned. The patients in the IC group who received PCE of 0-2 cycles, 3-4 cycles, 5-6 cycles, and 7-8 cycles were 1 (3.7%), 5 (18.5%), 5 (18.5%), and 16 (59.3%), respectively. The reasons for not completing full cycles of 8 weekly PCE therapy in 14 patients were either not apparent tumor shrinkage observed at interim response evaluation after completing four cycles of PCE (n=5), and any grade 3 adverse events (n=4), good tumor response after 7 cycles (n=2), and other reasons (n=3). The objective response rate (ORR) by IC-PCE was 55.6%, including six patients (22.2%) with complete response (CR), nine (33.3%) with partial response (PR), and four with progression disease (PD) (**Table 2**). The ORR for different cycles of IC-PCE was 0%, 60%, and 75% in 0-4 cycles, 5-6 cycles, and 7-8 cycles, respectively (**Supplementary Table 2**). Following IC-PCE, 24 of 27 patients (88.9%) proceeded to CRT, while the remaining three received other therapies; all patients received systemic therapy or palliative RT for disease progression with distant metastasis. The other one with PD, who experienced progression of local disease progression but without distant disease progression, received CRT following the IC phase. Regarding compliance with CRT, %CRT completion was 93.3% in the CRT group and 83.3% in the patients treated with IC-PCE followed by CRT, with no significant difference between them (*p*=0.631). The patients who received cumulative CDDP of 0-100 mg/m2, 100-200 mg/m2, and >200 mg/m2 were 0 (0%), 1 (6.7%), and 14 (93.3%) patients in the CRT group and 1 (4.2%), 3 (12.5%), and 20 (83.3%) in the IC-PCE group, respectively. In the CRT group, one patient did not complete CRT due to grade 4 hyponatremia; of four patients in the IC-PCE group, the reasons for failure to complete CRT were either grade 3/4 hyponatremia, hypokalemia, or hypercalcemia in two patients, febrile neutropenia in one patient, and pneumonia in the other patient. At completion of CRT, ORR was 66.7% (CR: 60.0%, PR: 6.7%) in the CRT group and 95.8% (CR: 91.7%, PR: 4.2%) in the IC-PCE group, with this difference being statistically significant (*p*=0.024) (**Table 2**).

**Table 2:** Tumor response by RECIST ver.1.1.

	Number of patients (%)											
	Induction phase						CRT phase					
	CR	PR	SD	PD	NA	ORR	CR	PR	SD	PD	ORR	*p*-value^**^
**IC-PCE group** (n=27)	6 (22.2)	9 (33.3)	7 (25.9)	4 (14.8)	1 (3.7)	55.6%	22^*^ (91.7)	1^*^ (4.2)	0^*^ (0)	1^*^ (4.2)	95.8%	0.024
**CRT group** (n=15)	–	–	–	–	–		9 (60.0)	1 (6.7)	0 (0)	5 (33.3)	66.7%	

^*^Among 24 patients treated with IC-PCE followed by CRT. ^**^ t-test for ORR in CRT phase. Abbreviations: RECIST, Response Evaluation Criteria in Solid Tumors; CRT, chemoradiotherapy; PCE, paclitaxel, carboplatin and cetuximab; IC, induction chemotherapy; CR, complete response; PR, partial response; SD, stable disease; PD, progressive disease; NA, not available; ORR, objective response rate.

### 3.3 Prognosis

**Figure 2:**
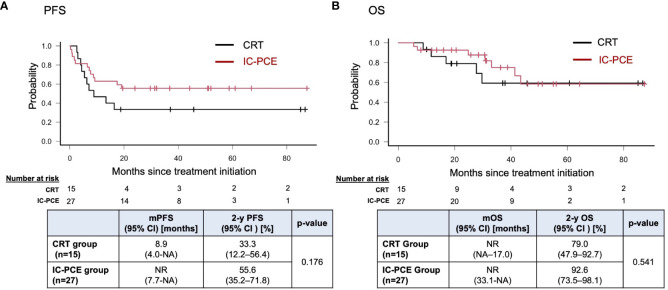
Progression-free survival **(A)** and overall survival **(B)** in the total population according to treatment. PFS, progression-free survival; mPFS, median PFS; OS, overall survival; mOS, median OS; NR, not reached; NA, not available.

**Figure 3:**
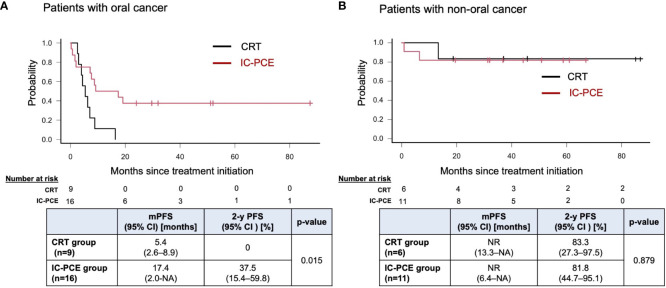
Progression-free survival in patients with oral cancer **(A)** and non-oral cancers **(B)** according to treatment. PFS, progression-free survival; mPFS, median PFS; NR, not reached; NA, not available.

With a median follow-up time of 31.2 months (range: 5.5 – 88.6) in the total population, the IC-PCE group showed a trend toward better PFS and OS than the CRT group (2-year PFS: 55.6% vs. 33.3%, log-rank *p*=0.176, 2-year OS: 92.6% vs. 79.0%, log-rank *p*=0.541) (**Figures 2A, B**). Next, we compared the subjects’ prognoses by clinical factors to extract the patient population which is most expected to benefit by adding IC-PCE to CRT. Among these, comparing prognoses in oral cancer and non-oral cancers in the CRT group revealed that the patients with oral cancer had a statistically significantly worse PFS and OS than those with non-oral cancers (**Supplementary Figure 2**). We then focused on analysis by tumor primary site (i.e., oral cancer vs. non-oral cancer), and as expected found that the significance of adding IC-PCE was more prominent and profound in patients with oral cancer, with the IC-PCE group showing better PFS than the CRT group (2-year PFS: 37.5% vs. 0%, log-rank *p*=0.015) (**Figure 3A**). On the other hand, the two groups showed an equally favorable prognosis for PFS in those patients with a non-oral primary cancer (**Figure 3B**). Regarding OS, the IC-PCE group showed a trend toward better survival than the CRT group (2-year OS: 87.5% vs. 63.5%, log-rank *p*=0.333) in those patients with oral cancer, versus no difference in those with non-oral cancer (2-year OS: 100% vs. 100%, log-rank *p*=1.000) (**Supplementary Figure 3**). Furthermore, we analyzed the impact of tumor responsiveness to IC on subsequent prognosis and found that the IC responders had more favorable PFS and OS than the non-responder group (2-year PFS: 80% vs. 18.2%, log-rank p=0.001, and 2-year OS: 100% vs. 81.8%, log-rank p=0.033), suggesting that the chemo-responsiveness of IC would be a prognostic factor in this clinical setting (**Supplementary Figure 4**).

### 3.4 Adverse events

Acute toxicities experienced during IC-PCE or CRT in the two groups are listed in **Tables 3** and **4**, respectively. Common grade 3/4 adverse events during IC-PCE were neutropenia (18.6%), leukopenia (3.7%), and acneiform dermatitis (11.1%). Notably, one patient experienced a grade 3 infusion reaction due to cetuximab; this patient could not continue PCE and was moved to CRT. The total frequency of grades 3/4 toxicity in the period of IC was 44.4% (**Table 3**), while the toxicities did not hamper proceeding to CRT in all cases. Regarding toxicities observed during CRT, mucositis (CRT group vs. IC-PCE group: 46.7% vs. 33.3%), leukopenia (20.0% vs. 20.8%), neutropenia (6.7% vs. 20.8%), and anemia (13.3 vs. 20.8%) were the most frequently observed grade 3/4 adverse events during CRT. The total frequency of grade 3/4 toxicity during CRT in the CRT and IC-PCE groups was 66.7% and 62.5%, respectively (**Table 4**). There was no treatment-related death throughout treatment in any case. As for late toxicity, no treatment-related deaths, including fatal bleeding, were observed.

**Table 3:** Selected toxicity during induction chemotherapy.

Toxicity	Number of patients (%)		
	All grades	Grade 3	Grade 4
Hematological toxicity			
Leukopenia	24 (88.9)	1 (3.7)	0 (0)
Neutropenia	22 (81.5)	5 (18.6)	0 (0)
Febrile neutropenia	0 (0)	0 (0)	0 (0)
Anemia	16 (59.3)	0 (0)	0 (0)
Thrombocytopenia	0 (0)	0 (0)	0 (0)
Non-hematological toxicity			
Infusion reaction	3 (11.1)	1 (3.7)	0 (0)
AST elevation	6 (22.2)	0 (0)	0 (0)
ALT elevation	12 (44.4)	0 (0)	0 (0)
Hyponatremia	3 (11.1)	3 (11.1)	0 (0)
Hypomagnesemia	13 (48.1)	0 (0)	0 (0)
Nausea	4 (14.8)	0 (0)	0 (0)
Dysgeusia	3 (11.1)	0 (0)	0 (0)
Mucositis	9 (33.3)	0 (0)	0 (0)
Anorexia	9 (33.3)	2 (7.4)	0 (0)
Fatigue	6 (22.2)	0 (0)	0 (0)
Peripheral neuropathy	7 (25.9)	0 (0)	0 (0)
Alopecia	6 (22.2)	0 (0)	0 (0)
Acneiform dermatitis	17 (63.0)	3 (11.1)	0 (0)
Other skin reaction^*^	16 (59.3)	0 (0)	0 (0)
Diarrhea	4 (14.8)	1 (3.7)	0 (0)
Constipation	4 (14.8)	0 (0)	0 (0)
**Total**	27 (100)	12 (44.4)	

ALT, alanine aminotransferase; AST, aspartate amino transferase. ^*^Including paronychia, dry skin, and skin cracks.

**Table 4:** Selected toxicity during chemoradiotherapy in the CRT group and IC-PCE group.

Toxicity	Number of patients (%)			
	All grades		Grade 3/4	
	CRT group (n=15)	IC-PCE group (n=24)	CRT group (n=15)	IC-PCE group (n=24)
Hematological toxicity				
Leukopenia	11 (73.3)	20 (83.3)	3 (20.0)	5 (20.8)
Neutropenia	9 (60.0)	17 (70.8)	1 (6.7)	5 (20.8)
Febrile neutropenia	0 (0)	1 (4.2)	0 (0)	1 (4.2)
Anemia	14 (93.3)	22 (91.7)	2 (13.3)	5 (20.8)
Thrombocytopenia	6 (40.0)	8 (33.3)	0 (0)	0 (0)
Non-hematological toxicity				
AST elevation	3 (20.0)	5 (20.8)	0 (0)	0 (0)
ALT elevation	5 (33.3)	6 (25.0)	1 (6.7)	0 (0)
Serum creatinine elevation	4 (26.7)	6 (25.0)	0 (0)	0 (0)
Hyponatremia	5 (33.3)	12 (50.0)	1 (6.7)	4 (16.7)
Hyperkalemia	3 (20.0)	4 (16.7)	0 (0)	0 (0)
Hypokalemia	1 (6.7)	2 (8.3)	0 (0)	2 (8.3)
Hypomagnesemia	3 (20.0)	8 (33.3)	0 (0)	0 (0)
Nausea	6 (40.0)	6 (25.0)	0 (0)	0 (0)
Dysgeusia	11 (73.3)	18 (75.0)	0 (0)	0 (0)
Mucositis	14 (93.3)	21 (87.5)	7 (46.7)	8 (33.3)
Dry mouth	9 (60.0)	13 (54.2)	0(0)	0 (0)
Anorexia	10 (66.7)	11 (45.8)	3 (20.0)	0 (0)
Fatigue	3 (20.0)	5 (20.8)	0 (0)	0 (0)
Constipation	7 (46.7)	9 (37.5)	0 (0)	0 (0)
Radiation dermatitis	12 (80.0)	5 (20.8)	0(0)	1 (4.2)
**Total**	15 (100)	24 (100)	10 (66.7)	15 (62.5)

ALT, alanine aminotransferase; AST, aspartate amino transferase.

## 4 Discussion

The current study evaluated the significance of IC in patients with unresectable locoregional recurrent SCCHN after curative surgery by comparing patients receiving IC followed by CRT with those receiving CRT alone. The results revealed that adding IC-PCE to CRT provided a promising improvement in prognosis, particularly in patients with oral cancer (2-year PFS: 37.5% vs. 0%, log-rank *p*=0.015), without any apparent increase in toxicity during CRT.

Among head and neck cancers, oral cancer has been recognized as a surgical disease, if applicable, primarily due to its relatively low sensitivity to chemotherapy and radiotherapy (14–17). In the clinical setting of initial curative treatment, the prognosis of patients with locally advanced oral cancer who harbor resectable disease but receive CRT is poor compared with that of patients treated with surgery plus postoperative (chemo) radiotherapy (17–19). A report based on the National Cancer Database of the United States revealed that surgery with postoperative radiotherapy provided a better prognosis than CRT in a propensity score-matched cohort (3-year OS: 51.8% vs. 39.3%) (20). This phenomenon was similarly seen in a population which developed local recurrence after curative surgery: the prognosis of patients who did not undergo salvage surgery was again reportedly poor (median OS: 5 months (5), 2-year OS: 0-20% (6, 21)), similar to the results seen in our patients with oral cancer treated with CRT alone (2-year PFS of 0% and 2-year OS of 63.5% in **Supplementary Figure 2**). Thus, a novel approach to improving the dismal prognosis of this population has been warranted.

Induction chemotherapy, which is usually prescribed prior to definitive CRT in attempting to improve prognosis in patients whose prognosis is strongly expected to be unsatisfactory with CRT alone, has been tested in locally advanced head and neck cancer, including oral cavity cancer. However, the representative triplet regimen (i.e., TPF) has failed to show any value, at least in the initial curative setting (8–10). Allowing that the percentage of patients with oral cavity cancer in these previous studies was generally low (14-21%), no study has yet evaluated the significance of IC in patients with unresectable local recurrence after curative surgery. Against this background, we believe our current findings of the potential clinical significance of IC-PCE prior to CRT would be of benefit in the patient population whose prognosis is disappointingly poor with CRT alone (**Supplementary Figure 2**).

As for the reported efficacy of IC-PCE in the current study, 55.6% of ORR was relatively lower than previously reported (91%) in patients with untreated locally advanced SCCHN (22). We speculate that this is likely due to the highly aggressive disease feature causing recurrence after curative resection. Since no patient received chemotherapy even as postoperative chemoradiotherapy before treatment, we could not assess the potential impact of pretreatment systemic therapy on the observed relatively low ORR. On the other hand, although approximately 20% of patients obtained CR after IC-PCE in the current study, chemoradiotherapy, not surgery, was given as planned. Indeed, several reports suggested that the chemotherapy responder may have a favorable prognosis by subsequent surgery and postoperative chemoradiotherapy in unresectable but previously untreated head and neck cancer (23, 24). However, in the population with unresectable loco-regional recurrence after curative surgery, even if radiological CR is obtained, complete surgical resection is usually technically challenging, and we thus believe chemoradiotherapy should be practical and reasonable in this situation.

Concerning prognosis, we must consider systemic therapy, a usually selected treatment for patients with unresectable recurrent squamous cell carcinoma of the head and neck, as a competitive one to the RT-containing strategy discussed in the current study. Especially pembrolizumab-containing systemic therapy has been recognized as standard systemic therapy for platinum-sensitive recurrent or metastatic disease based on the results of Keynote-048 (25). In this setting, we recognized that the data on PFS, which is one of the most prioritized outcomes, is numerically favored toward IC-CRT reported in the current study when compared with pembrolizumab plus chemotherapy in Keynote-048 (2y-PFS: 55.6% vs.10.7%). Moreover, OS as an ultimate outcome is also better in the IC-CRT (2y-OS: 92.6% vs. 29%). Based on these findings, although we cannot reach a decisive conclusion, we believe the RT-containing strategy, especially IC-CRT, should be a promising therapeutic option in anticipating better clinical outcomes, including cure, if applicable.

We are unable to explain the improvement in results with the addition of IC-PCE in the oral cavity population. One possible explanation may be cetuximab’s targeting of EGFR as a part of PCE and the characteristic nature of oral cavity cancer relating to EGFR. An analysis of the gene expression profile of head and neck cancer found that a phenotype called the basal type, in which gene expression of the pathway related to *EGFR* is activated, is particularly common in oral cancer (26). Further, *in vitro* studies have shown that *EGFR* is abundantly expressed on oral cancer cells and that cetuximab-mediated antibody-dependent cellular cytotoxicity (ADCC) is a crucial process associated with the therapeutic efficacy of cetuximab (27–29).

Clinically, the EXTREME trial, which evaluated the effect of platinum–fluorouracil plus cetuximab in patients with recurrent or metastatic squamous cell carcinoma of the head and neck, suggested that cetuximab was preferentially effective in the oral cancer population, with a PFS-hazard ratio (HR) favoring chemotherapy plus cetuximab (PFS-HR: 0.34 [95%CI: 0.21-0.55]) compared with chemotherapy plus placebo (30). Moreover, two randomized trials (SPECTRUM and ZALUTE) which evaluated different anti-EGFR monoclonal antibodies (panitumumab and zalutumumab, respectively) also showed preferential antitumor effects compared with their control arms in patients with primary oral cancer (PFS-HRs: 0.70 [95%CI: 0.51-0.96] and 0.54 [0.31-0.93], respectively) (31–33).

A limitation of this study is that it was a retrospective evaluation of a small number of patients from a single institution. No prospective comparison with CRT alone has yet appeared, however. Confirmation of the role of PCE as induction chemotherapy in these patients requires a multicenter case series or prospective randomized controlled trial with a large sample size. Besides, because the current study focuses on patients who did not receive postoperative (chemo)radiotherapy as part of consecutive initial curative treatment, we could not address whether the current proposed treatment strategy with IC-PCE followed by CRT truly overcome the dismal prognosis, which might be avoided by performing postoperative (chemo)radiotherapy. Also, our present study did not assess whether other IC, such as TPF as a non-cetuximab-containing regimen, would provide similar clinical significance. Notably, there has been discussion on developing a practical and feasible IC regimen because its toxicity often compromises compliance with subsequent local therapy here in CRT. For example, a modified TPF regimen in which relatively low administrative doses are adopted retained favorable ORR (89.6%) and more manageable safety profiles than conventional TPF in the initial treatment setting (34). Another example is IC with Docetaxel + CDDP + Cmab (TPEx), characterized by replacing 5-FU with Cmab, which also showed reliable ORR (72.2%) in the same setting (35). Thus, this unanswered question should also be addressed in future studies seeking ideal IC regimens as non-surgical curative treatment.

## 5 Conclusion

In patients with squamous cell carcinoma of the head and neck with unresectable locoregional recurrence after curative surgery and no history of radiation, induction chemotherapy with PCE before CRT may improve prognosis, especially in patients with oral cancer.

## Data availability statement

The raw data supporting the conclusions of this article will be made available by the authors, without undue reservation.

## Ethics statement

The studies involving humans were approved by Institutional Review Board of the National Cancer Center Hospital East (2016-243). The studies were conducted in accordance with the local legislation and institutional requirements. Written informed consent for participation was not required from the participants or the participants’ legal guardians/next of kin in accordance with the national legislation and institutional requirements.

## Author contributions

MS: Conceptualization, Data curation, Formal analysis, Writing – original draft, Writing – review & editing. TE: Conceptualization, Writing – original draft, Writing – review & editing, Data curation, Formal analysis. TF: Conceptualization, Data curation, Writing – review & editing. SO: Data curation, Writing – review & editing. NaT: Data curation, Writing – review & editing. NoT: Data curation, Writing – review & editing. HT: Data curation, Writing – review & editing. AM: Data curation, Writing – review & editing. SZ: Data curation, Writing – review & editing. TS: Data curation, Writing – review & editing. KM: Data curation, Writing – review & editing. RH: Data curation, Writing – review & editing. TA: Data curation, Writing – review & editing. MT: Conceptualization, Writing – original draft, Writing – review & editing.

## Supplementary material

Supplementary Figure 1 Patient extraction process. ^*^One patient who received IC-PCE followed by RT alone was excluded from the study because of intolerance to cisplatin due to renal dysfunction prior to the start of treatment. RT, radiotherapy; 5-FU, 5-fluorouracil; CBDCA, carboplatin; TPS, docetaxel + cisplatin + S-1; CDDP, cisplatin; PCE, paclitaxel + carboplatin + cetuximab; CRT, chemoradiotherapy; IC, induction chemotherapy.

Supplementary Figure 2 Progression-free survival **(A)** and overall survival **(B)** in the CRT group according to treatment. PFS, progression-free survival; mPFS, median PFS; OS, overall survival; mOS, median OS; 2-y OS, two-year OS; NR, not reached; NA, not available.

Supplementary Figure 3 Overall survival in patients with oral cancer **(A)** and non-oral cancers **(B)**. OS, overall survival; mOS, median OS; NR, not reached; NA, not available.

Supplementary Figure 4 Progression-free survival **(A)** and overall survival **(B)** in the IC-PCE group according to response to IC. PFS, progression-free survival; mPFS, median PFS; OS, overall survival; mOS, median OS; 2-y OS, two-year OS; NR, not reached; NA, not available.
