# Supplementary material for: Induction chemotherapy with paclitaxel, carboplatin, and cetuximab (PCE) followed by chemoradiotherapy for unresectable locoregional recurrence after curative surgery in patients with squamous cell carcinoma of the head and neck
Source: Front Oncol. 2024 Jul 1;14:1420860. doi: 10.3389/fonc.2024.1420860 (PMC11246904; doi:10.3389/fonc.2024.1420860)
Supplement: Supplementary file 1 [file Table_1.docx]

**Supplementary Table 1. Patient characteristics**

|  | **CRT group, n=15** | **IC-PCE group, n=27** | **p-value** |
| --- | --- | --- | --- |
| **cT stage at last recurrence** |  |  |  |
| rT0 | 9 (60.0) | 17 (63.0) | 0.938 |
| rT1 | 0 | 1 (3.7) |  |
| rT2 | 1 (6.7) | 2 (7.4) |  |
| rT3 | 1 (6.7) | 3 (11.1) |  |
| rT4a | 2 (13.3) | 2 (7.4) |  |
| rT4b | 2 (13.3) | 2 (7.4) |  |
| **cN stage at last recurrence** |  |  |  |
| rN0 | 5 (33.3) | 5 (18.5) | 0.634 |
| rN1 | 2 (13.3) | 2 (7.4) |  |
| rN2b | 4 (26.7) | 8 (29.6) |  |
| rN2c | 3 (20.0) | 6 (22.2) |  |
| rN3b | 1 (6.7) | 6 (22.2) |  |
| **cTN stage at last recurrence** |  |  |  |
| rT0N1 | 2 (13.3) | 1 (3.7) | 0.749 |
| rT0N2/3 | 7 (46.7) | 16 (59.3) |  |
| rT1/2N0 | 1 (6.7) | 1 (3.7) |  |
| rT1/2N2/3 | 0 (0) | 2 (7.4) |  |
| rT3/4N0 | 4 (26.7) | 4 (14.8) |  |
| rT3/4N1 | 0 (0) | 1 (3.7) |  |
| rT3/4N2/3 | 1 (6.7) | 2 (7.4) |  |
| **Reasons for deeming the disease unresectable** |  |  |  |
| Difficulty in performing surgical resection | 5 (33.3) | 13 (48.1) | 0.517 |
| Inability to control the disease by surgical resection^*^ | 10 (66.7) | 14 (51.9) |  |

^*^ The inability to control tumor progression by surgical resection was defined by early and rapidly progressed locoregional recurrence revealed by planned CT before postoperative radiotherapy or far advanced lymph node metastasis (e.g., retropharyngeal [so-called Rouviere], mediastinal or supraclavicular lymph nodes). Abbreviations: RT, radiotherapy; IC, induction chemotherapy.
