# Supplementary material for: Induction chemotherapy with paclitaxel, carboplatin, and cetuximab (PCE) followed by chemoradiotherapy for unresectable locoregional recurrence after curative surgery in patients with squamous cell carcinoma of the head and neck
Source: Front Oncol. 2024 Jul 1;14:1420860. doi: 10.3389/fonc.2024.1420860 (PMC11246904; doi:10.3389/fonc.2024.1420860)
Supplement: Supplementary file 2 [file Table_2.docx]

**Supplementary Table 2. Tumor response for IC-PCE by treatment cycles**

|  | | **Tumor response** | | | | | |
| --- | --- | --- | --- | --- | --- | --- | --- |
|  |  | **CR** | **PR** | **SD** | **PD** | **NA** | **ORR** |
| **# of PCE cycles completed** | **0-2** | 0 | 0 | 0 | 0 | 1 | 0% |
|  | **3-4** | 0 | 0 | 3 | 2 | 0 | 0% |
|  | **5-6** | 1 | 2 | 2 | 0 | 0 | 60% |
|  | **7-8** | 5 | 7 | 2 | 2 | 0 | 75% |

Abbreviations: PCE, paclitaxel, carboplatin and cetuximab; CR, complete response; PR, partial response; SD, stable disease; PD, progressive disease; NA, not available; ORR, objective response rate.
