# Supplementary figures and images for: Induction chemotherapy with paclitaxel, carboplatin, and cetuximab (PCE) followed by chemoradiotherapy for unresectable locoregional recurrence after curative surgery in patients with squamous cell carcinoma of the head and neck
Source: Front Oncol. 2024 Jul 1;14:1420860. doi: 10.3389/fonc.2024.1420860 (PMC11246904; doi:10.3389/fonc.2024.1420860)

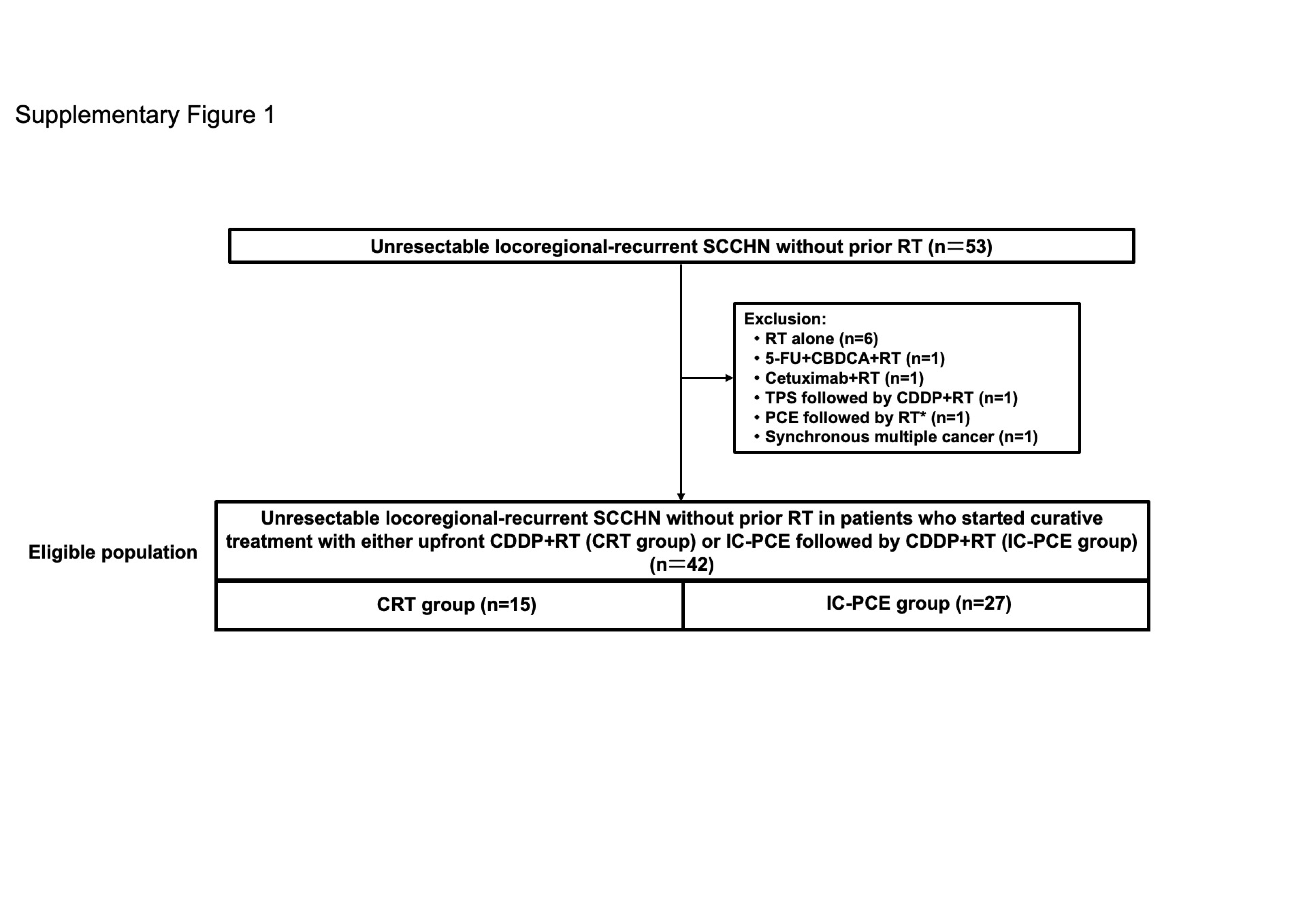

Supplement: Supplementary Figure 1 — Patient extraction process. *One patient who received IC-PCE followed by RT alone was excluded from the study because of intolerance to cisplatin due to renal dysfunction prior to the start of treatment. RT, radiotherapy; 5-FU, 5-fluorouracil; CBDCA, carboplatin; TPS, docetaxel + cisplatin + S-1; CDDP, cisplatin; PCE, paclitaxel + carboplatin + cetuximab; CRT, chemoradiotherapy; IC, induction chemotherapy. [file Image_1.jpeg]

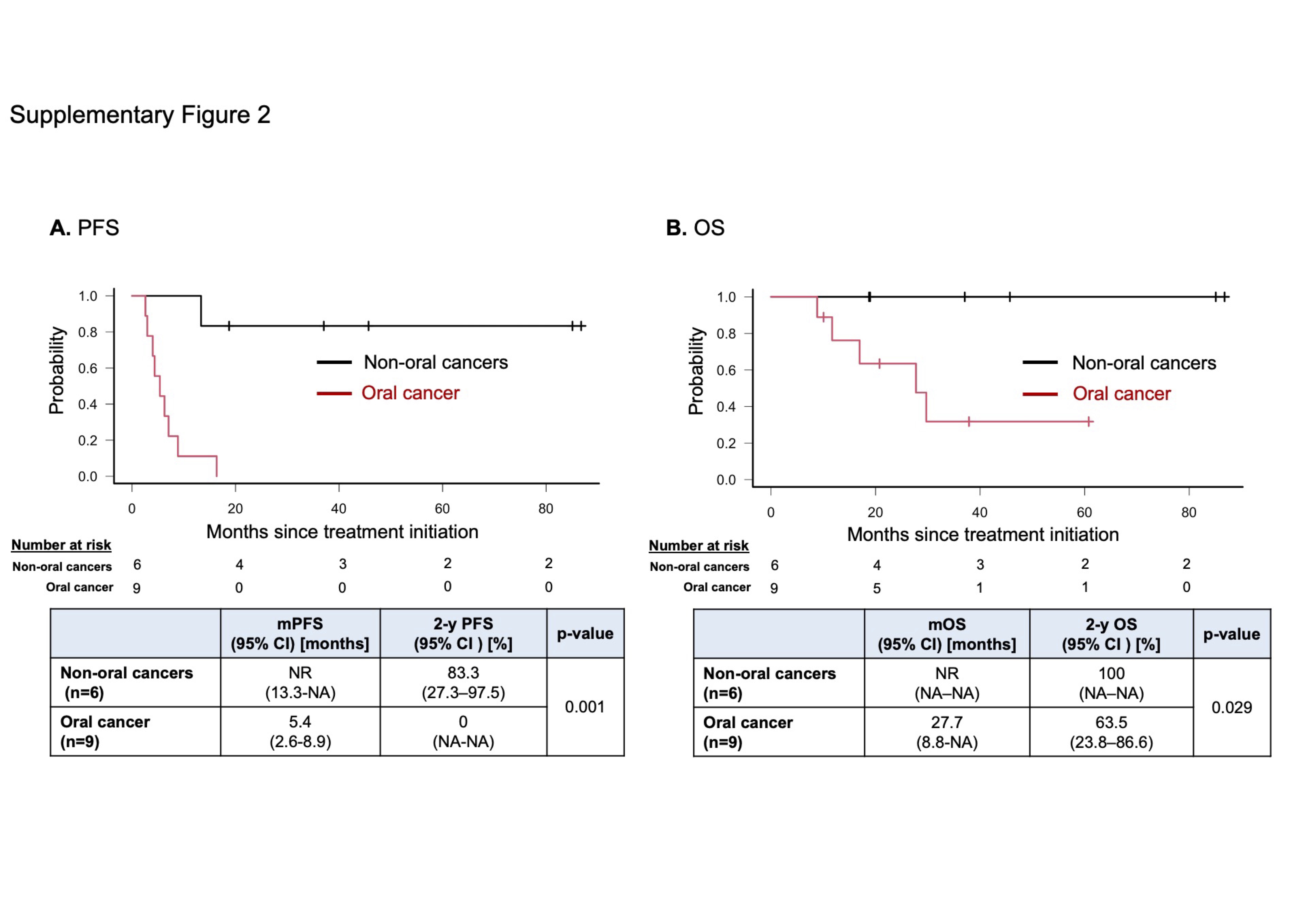

Supplement: Supplementary Figure 2 — Progression-free survival (A) and overall survival (B) in the CRT group according to treatment. PFS, progression-free survival; mPFS, median PFS; OS, overall survival; mOS, median OS; 2-y OS, two-year OS; NR, not reached; NA, not available. [file Image_2.jpeg]

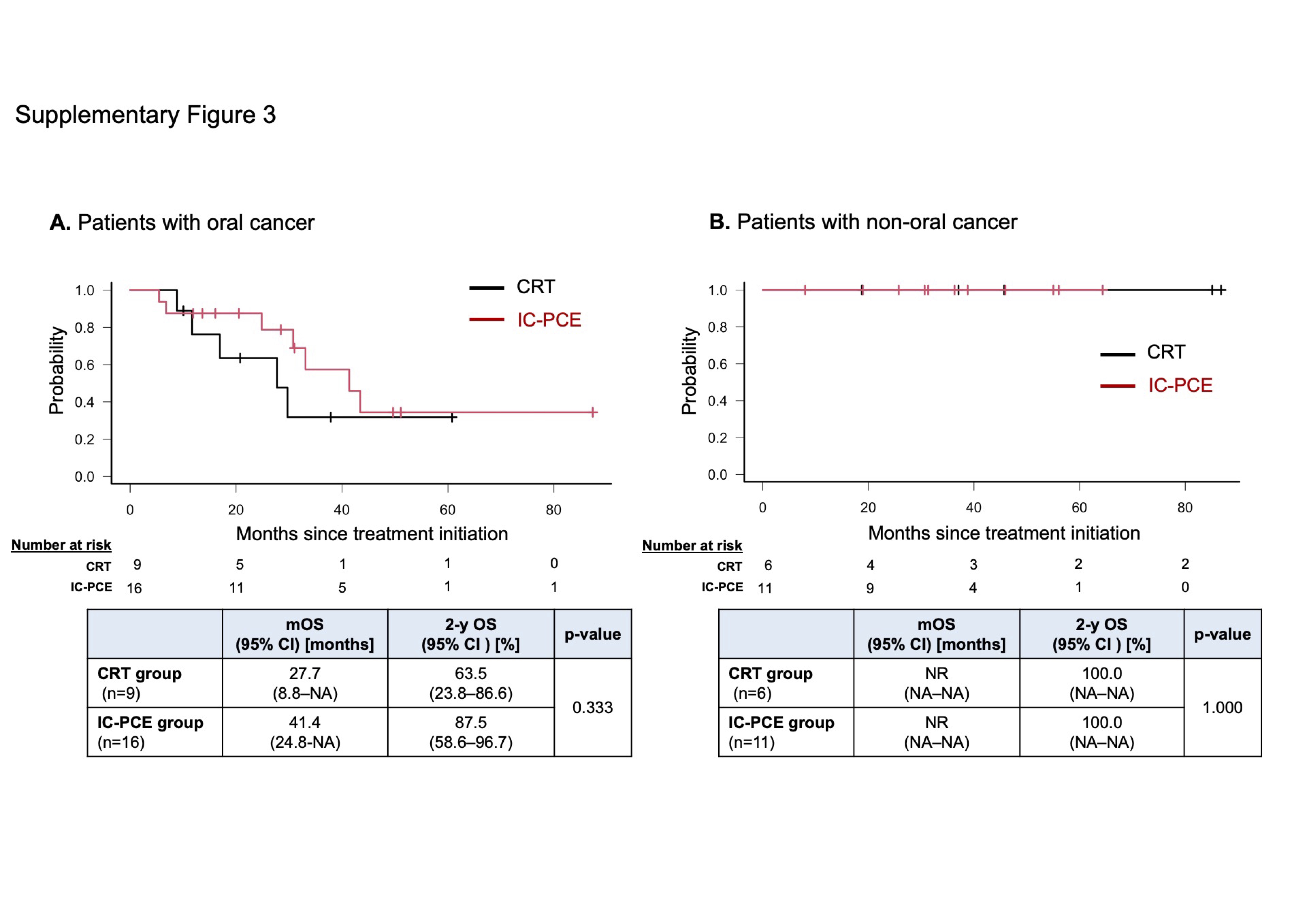

Supplement: Supplementary Figure 3 — Overall survival in patients with oral cancer (A) and non-oral cancers (B). OS, overall survival; mOS, median OS; NR, not reached; NA, not available. [file Image_3.jpeg]

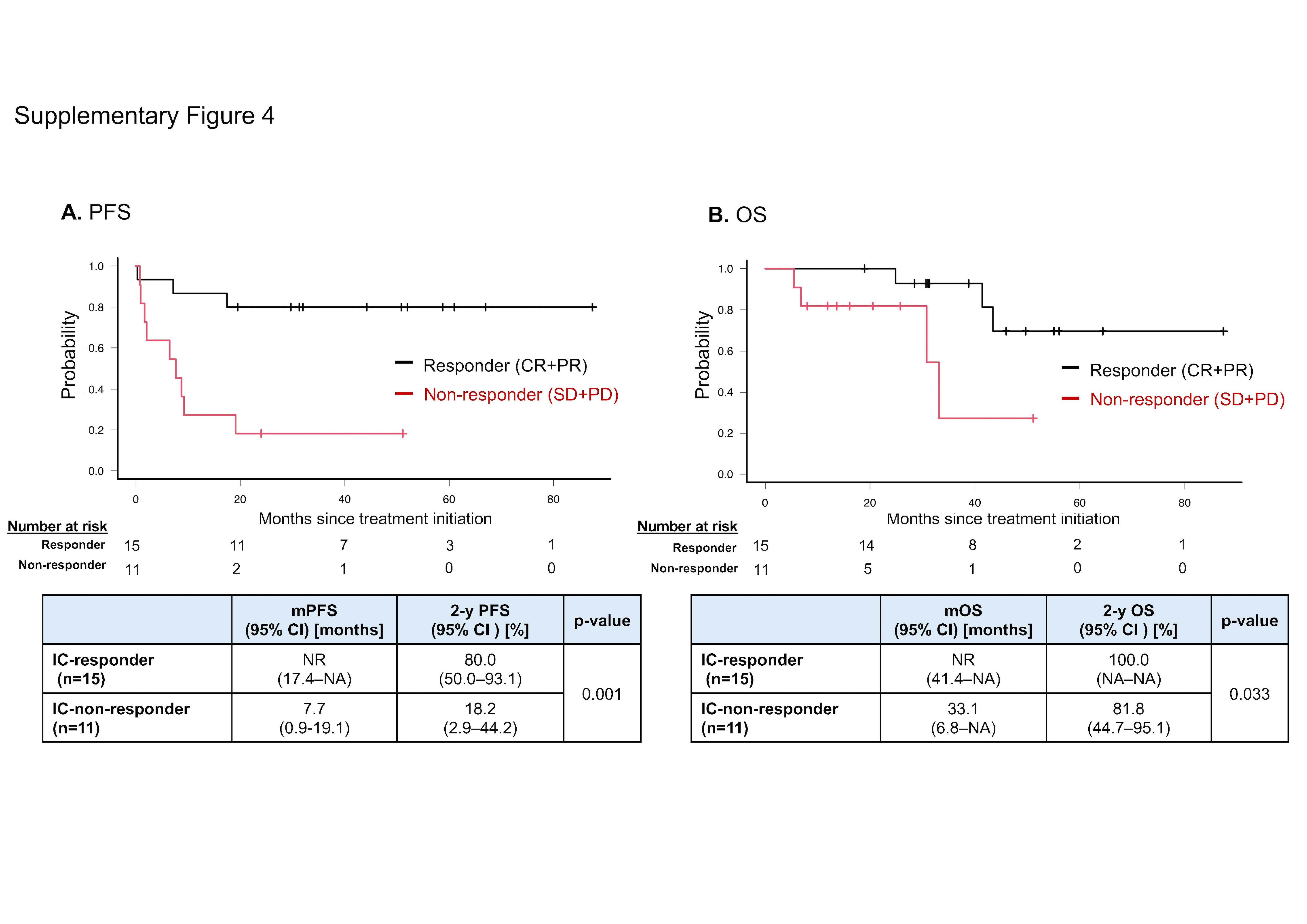

Supplement: Supplementary Figure 4 — Progression-free survival (A) and overall survival (B) in the IC-PCE group according to response to IC. PFS, progression-free survival; mPFS, median PFS; OS, overall survival; mOS, median OS; 2-y OS, two-year OS; NR, not reached; NA, not available. [file Image_4.jpeg]
